# New concept of scapholunate dissociation treatment and novel modification of Brunelli procedure - anatomical study

**DOI:** 10.1186/1471-2474-15-172

**Published:** 2014-05-23

**Authors:** Ahmed Elsaftawy, Jerzy Jabłecki, Tomasz Jurek, Adam Domanasiewicz, Bohdan Gworys

**Affiliations:** 1 St. Hediwg Hospital, Trzebnica, ul.Prusicka 53-55, 55-100 Trzebnica, Poland; 2 Department of Forensic Medicine Unit, Wroclaw Medical University, ul. J. Mikulicza-Radeckiego 4, 50-345 Wrocław, Poland; 3 Department of Anatomy, Wroclaw Medical University, ul. J. Mikulicza-Radeckiego 4, 50-345 Wrocław, Poland

**Keywords:** Scapholunate dissociation, Scapholunate instability, Modified Brunelli procedure, Rotatory subluxation of the scaphoid

## Abstract

**Backgrounds:**

This paper describes a novel method in treatment of scapholunate dissociation accompanied with rotatory subluxation of the scaphoid. The idea of this method is to create a kind of axial lever that can fully reconstruct anatomical relationship between the scaphoid and the lunate, with no involvement of extrinsic ligaments, and with simultaneous restriction of pathological alignment of the scaphoid. Based on this technique, we have also proposed a new modification of Brunelli procedure in scapholunate dissociation with rotatory subluxation of the scaphoid and dorsal intercalated segmental instability.

**Methods:**

At the initial stage of the study, 20 human wrists fixed in Ethanol were used, followed by 12 fresh human wrists used in part two. The first stage included functional, biomechanical and strength tests carried out by means of a 5 kg load and intended to find the most anatomical and durable treatment method. The second stage involved testing the proposed methods on fresh cadaver wrists.

**Results:**

We have discovered that the new method is able to recreate anatomical forces and properties of scapholunate ligament; what’s more, it can also prevent rotatory subluxation of the scaphoid. The performed strength tests have proven that it is possible to treat scapholunate instability also in case of dorsal intercalated segmental instability.

**Conclusions:**

We highly recommend using both the new technique and the new modification of Brunelli procedure for treatment of scapholunate dissociation in both dynamic and static instabilities.

## Background

**Figure 1:**
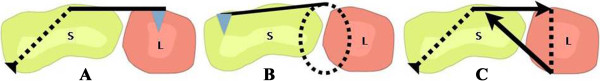
**The described three techniques of SLD treatment. A** - a diagram showing the idea of modified Brunelli technique using FCR tendon graft through a hole created in distal part of scaphoid (S), then FCR tendon is anchored to the lunate (blue triangle). **B** - new method of scapholunate tendon reconstruction – created perpendicular tunnels in the scaphoid and lunate through which a free tendon graft was introduced (the dotted line) and distally anchored to dorsal distal surface of the scaphoid in order to prevent its rotatory subluxation. **C** - new modification of Brunelli technique – after that FCR was introduced through the scaphoid, its introducing through the lunate hole from the side of midcarpal joint, and then tensioned and sutured to itself at scaphoid surface.

Scapholunate instability is the most frequent pattern of carpal instability occurring separately and as part of other wrist disorders. It may be classified as acute or chronic and static or dynamic, with or without dorsal intercalated segmental instability (DISI deformity). Scapholunate interosseous ligament (SLIL) has been indicated as the primary stabilizer of the scapholunate (SL) articulation, while the dorsoradial carpal ligament (DRCL), the dorsal intercarpal (DIC) ligament, the scaphotrapezial (ST) ligaments, and the radioscaphocapitate (RSC) ligaments have been shown as secondary stabilizers [1]. Injury to the dorsal part of SLIL alone does not create a typical static scapholunate dissociation (SLD) pattern with rotatory subluxation of the scaphoid; however, it may cause a dynamic instability [2,3]. On the other hand, a static scapholunate dissociation is unlikely to occur without disruption of palmar ligaments – RSC, with both long and short radiolunate (RL) ligaments [4,5] being affected. The dependence of DISI deformity on disruption of the DRCL [6] should also be taken into consideration. Although characteristics of these injuries are well known, their treatment is often a major challenge for many hand surgeons. This stems from the fact that many patients are diagnosed too late, like weeks, months or even years after sustaining the injury, with the instability having turned static already [7-9]. Chronic SLD has been used to describe ligament tears diagnosed over 6 weeks after injury. The greatest importance of chronicity of the injury is the potential healing of scapholunate ligament, whether the ligament can be directly repaired and whether the scaphoid is reducible. There are many techniques to treat chronic dynamic and static scapholunate instability, starting from arthroscopic methods, via scapholunate ligament primary repair, repair with various types of capsulodesis, repair with tendon graft reconstruction, bone-ligament-bone procedure, and ending with various intercarpal fusions [10-14]. According to Garcia-Elias [15], if there are no signs of intercarpal arthritis, it is crucial that we determine answers to several questions: can the ligament be repaired primarily?, is the rotatory subluxation of the scaphoid present? and if so- can the scaphoid be reduced easily? Are there any signs of DISI deformity? Several procedures have been designed to treat and restrict rotatory subluxation of the scaphoid in case of chronic instabilities. As far as open surgery patterns are considered, the most commonly used methods are the modified Brunelli and the three-ligament tenodesis by Garcia-Elias [Figure 1].

This paper aims at presenting a novel method of free tendon reconstruction of scapholunate ligament complex, which proves particularly useful on scapholunate ligament dissociation with rotatory subluxation of the scaphoid. We also propose a new modification of Brunelli procedure in scapholunate dissociation with rotatory subluxation of the scaphoid and dorsal intercalated segmental instability (Figure 1).

## Methods

The first part of the study involved 20 human wrists fixed in Ethanol, while at the second stage 12 fresh human wrists were used (whose donors had voluntarily donated their bodies for scientific purposes after their demise). The proposed method of free tendon reconstruction of scapholunate ligament complex involves formation of a stable axial lever for the scaphoid and the lunate, which restores the anatomical pattern of scapholunate ligament complex as well as prevents occurrence of rotatory subluxation of the scaphoid.

**Figure 2:**
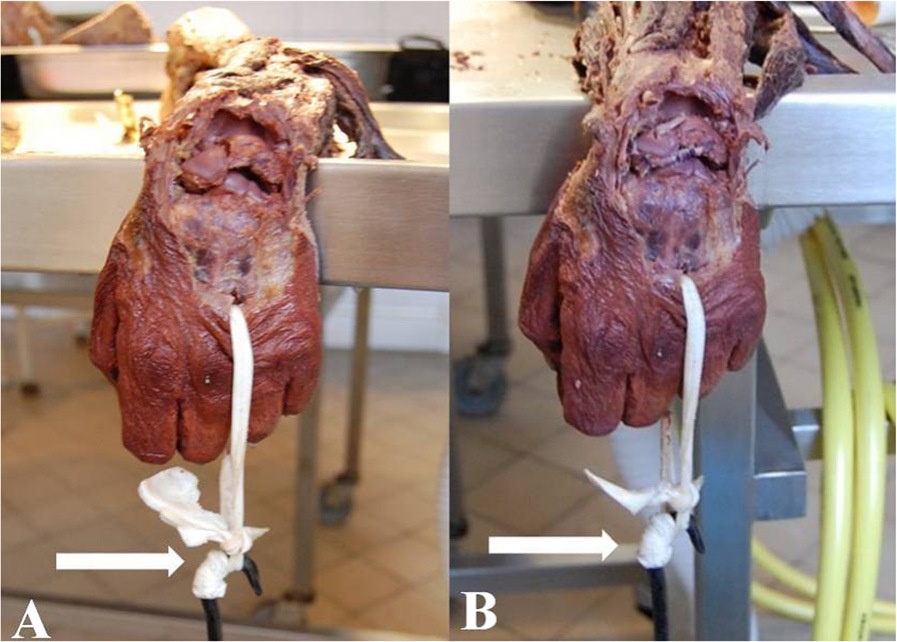
**Strength tests with 5 kg load (white arrows). A** - after intentional damage to all primary and secondary stabilizers of scapholunate ligament complex, **B** - after applying new technique of free tendon reconstruction.

**Figure 3:**
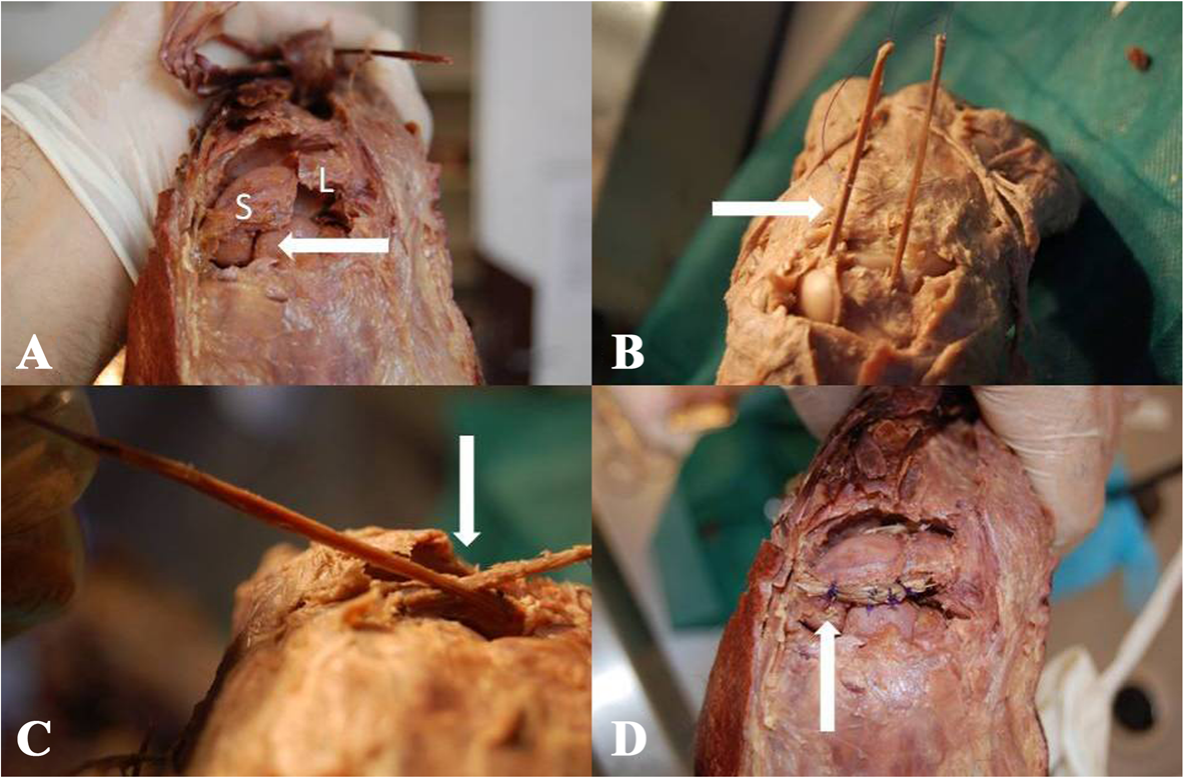
**Demonstration of the new technique. A** - scapholunate dissociation with rotatory subluxation of the scaphoid (white arrow). S – scaphoid, L – lunate. **B** - free tendon graft is introduced through tunnels created in the scaphoid and the lunate (white arrow). **C** - when carpal bones were correctly aligned, the tendon graft was interlaced (white arrow). **D** - tendon graft was sutured at the intersection point and it’s both sides. Lunate part of the tendon was then distally anchored to the dorsal distal surface of the scaphoid in order to prevent its rotatory subluxation.

**Figure 4:**
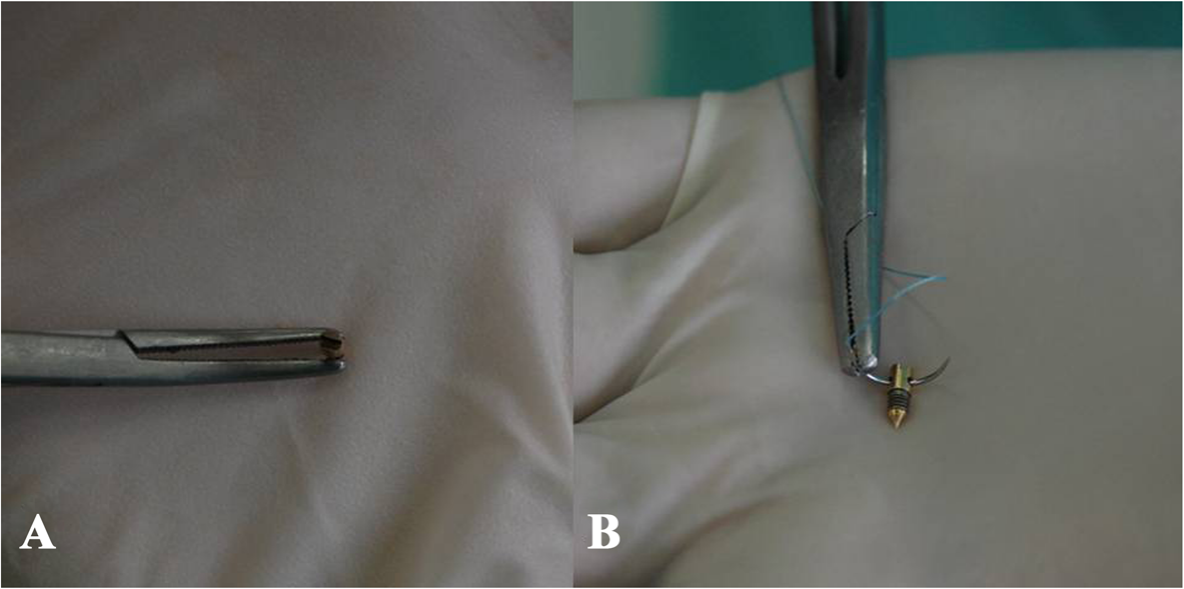
**The anchors design that have been used at the study. A** - It can be easily screwed in the bone. **B** - It has a hole through which a suture material could be introduced.

**Figure 5:**
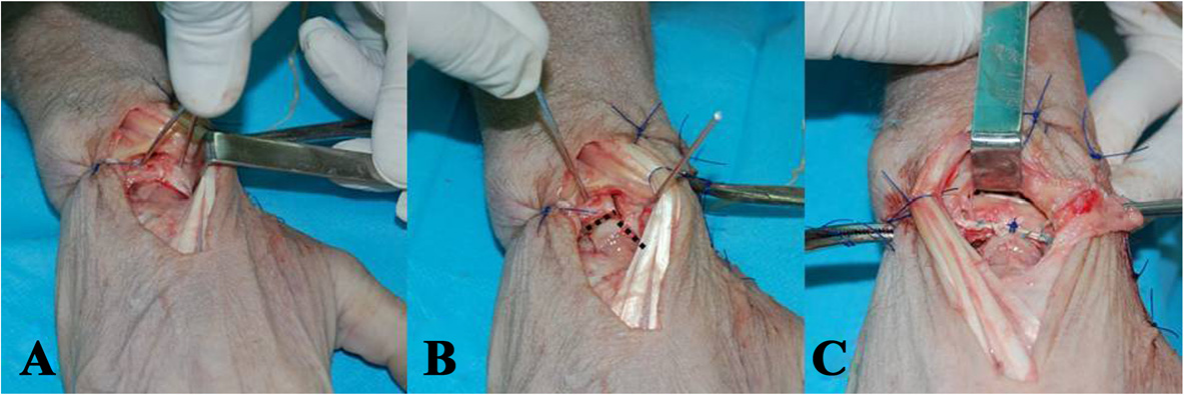
**The second part of the study, the new method was tested on fresh cadaver wrists. A** - intentional damage to all elements of scapholunate complex was inflicted. **B** - scapholunate dissociation is shown with scaphoid malalignment (dashed black line). **C** - scapholunate ligament complex reconstructed with the new technique

**Figure 6:**
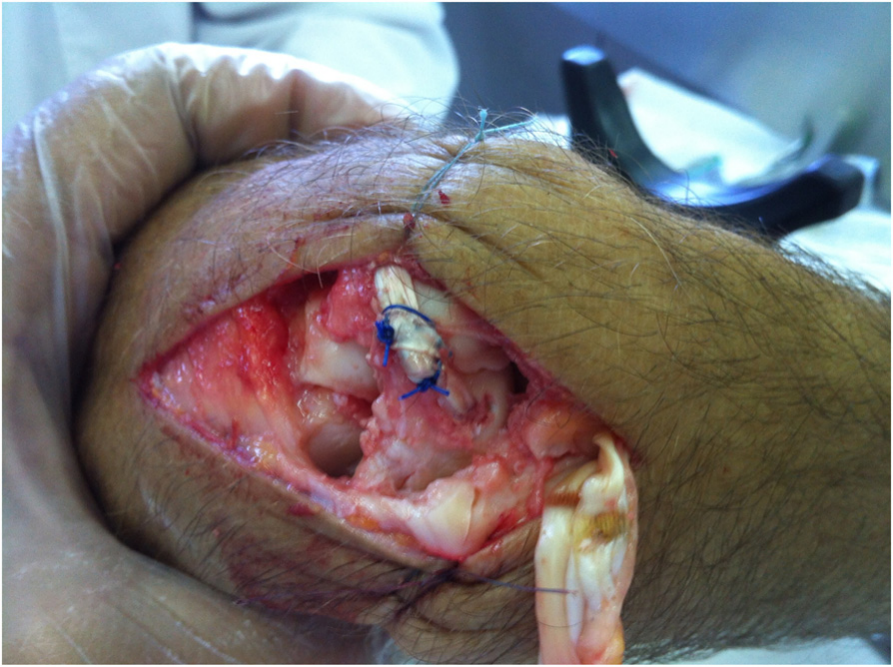
New modification of Brunelli procedure – FCR tendon introduced through an oblique tunnel in the scaphoid, then its introduction through the lunate tunnel from the side of midcarpal joint and afterwards its tensioning and suturing to itself at the scaphoid surface.

The initial stage included functional, biomechanical and strength tests performed in order to find out if the new treatment method, which can be used without involvement of extrinsic carpal ligaments that might limit wrist mobility, could effectively restore the anatomical integrity of scapholunate complex. An intentional damage to the primary (SLIL) and secondary stabilizers (RSC, DRCL, DIC and ST ligaments) of the SL ligament complex was inflicted. Each modification has been examined in terms of mechanical properties and strength by means of 5 kg test load swinging back and forth (Figure 2). We measured the gap between the scaphoid and the lunate, as well as the difference in scaphoid shift in relation to lunate surface from the side of midcarpal joint. Measurements were taken from dorsal aspect of the wrists. The results were then compared to the states before and after the procedures were performed. The idea was to create a perpendicular tunnels of 2.5 mm diameter in the scaphoid and the lunate, through which a free tendon graft was introduced. The axial lever that fully reconstructs the anatomical relationship between scaphoid and lunate has been found to be the best option to restore the integrity of scapholunate complex. The drilling began from the intercarpal joint side at 30° angle at the interface between radiocarpal and midcarpal joints. The most optimal position for drilling the tunnels in the scaphoid and lunate was about 3 mm from each side of scapholunate joint. We encountered a problem at distal part of the scaphoid which when unfastened, could easily result in rotatory subluxation of the scaphoid if secondary stabilizers were disrupted. This is why special care was given to ensure that the lunate part of the graft is twice as long as the scaphoid one. Once carpal bones were correctly aligned (via “K” wire fixation through the scapholunate – 2 “K” wires – and scaphocapitate – 1 “K” wire – joints), the tendon graft was interlaced and sutured at the intersection point and its both sides over the lunate and proximal pole of the scaphoid. The aforementioned lunate part of the tendon was then distally anchored to the dorsal distal surface of the scaphoid in order to prevent its rotatory subluxation (Figure 3). Especially for the study, we designed and produced new anchors, which had a hole inside, through which a suture material could be introduced. We were able to screw in the anchors as it is shown in Figure 4. At the second stage of the study the proposed method was tested on fresh cadaver wrists with palmaris longus free tendon graft (6 fresh cadaver wrists) and compared with modified Brunelli procedures by means of flexor carpi radialis (FCR) tendon (3 fresh cadaver wrists) (Figure 5). The strength of the procedures was tested under a 5 kg load when the wrist was in neutral position and flexion. We also tried a new modification for Brunelli procedure according to our own technique (3 fresh cadaver wrists). After introducing a slip of FCR tendon through the scaphoid, we created a new tunnel in the lunate, like it was done in the method mentioned earlier. After that, FCR slip was introduced through this tunnel from the side of midcarpal joint, and then tensioned and sutured to itself at the scaphoid surface (Figure 6). In this modification, FCR tendon will pull up the lunate so that it is in greater flexion than extension. This can be achieved in cases with DISI deformity present. Our research complies with the Declaration of Helsinki and has been approved by the Commission of Bioethics at Wroclaw Medical University – reference number KB – 894/2012.

## Results

While an average gap width after sectioning of the SLIL and the secondary stabilizers of scapholunate complex amounts to 2,59 mm (range 2–3 mm) under 5 kg load, after applying the new procedures it was only 1,31 mm (range 1–2 mm). The average difference of scaphoid shift in relation to the lunate surface from the side of midcarpal joint used to be 8,1 mm (range 6–12 mm) and we managed to reduce it to 2,62 mm (range 1–4 mm) by means of the new procedures. The method discussed withstood 5 kg load with no signs of scapholunate dissociation or rotatory subluxation of the scaphoid. The set p-value was less than 0.05 (0.00078 and 0.00279 respectively). Thus, the comparison of the two features had to be performed by a paired Wilcoxon’s test. In both cases, the differences between groups were significant. The p-value for this test was 5.96-08 (for reducing the SL gap), and for prevention rotatory subluxation of the scaphoid was 3.73-09.

To prevent the gap from increasing between the scaphoid and the lunate when applying this method, the tendon graft in place of intersection has to be sutured to itself in three points: at the intersection point and its both sides over the lunate and proximal pole of scaphoid. The shape and the way of fastening of the new anchors were very helpful due to the possibility of their multiple use.

An average gap width after modified Brunelli procedure was 1 mm (range 1–2 mm). After the modified Brunelli procedure was performed, average difference of scaphoid shift in relation to the lunate surface from the side of midcarpal joint was 2,33 mm (range 2–3 mm). Since in this group there were only 3 cases it was decided that a comparison of unpaired Wilcoxon’s test should be performed. In both cases, the differences between groups were significant. The p-value for this test was 0,00145 for reducing the gap between scaphoid and lunate, and for prevention rotatory subluxation of the scaphoid was 0,00402.

The modified Brunelli procedure seemed to both reduce the SL gap and prevent rotatory subluxation of the scaphoid, yet it limited wrist flexion movements because of involvement of dorsal extrinsic ligaments. The average angle of maximum wrist flexion after applying modified Brunelli procedure was 53.3° (range 50°-60°), however when we attempted to obtain a greater flexion by applying more strength, the place of suturing FCR to itself started to break in all three cases. The new modification of Brunelli procedure was more stable, with no evidence of scapholunate dissociation or scaphoid subluxation.

## Discussion

Carpal instability with some of its clinical manifestations had been described by Destot [16] in 1926, but the term „instability” was introduced by Dobyns [17] in 1967. In scapholunate dissociation the capitate migration down into the scapholunate joint tends to create a gap between the scaphoid and the lunate. This will cause extension of the lunate and flexion of the scaphoid, giving rise to an increased scapholunate angle as a result of scapholunate interosseous ligament attenuation. This is also accompanied by an increased capitolunate angle. An additional factor is the increased ulnar translation of the lunatotriquetral complex due to the release of the scapholunate interosseous ligament. Acute injuries can practically always be improved without surgery if standard x-rays and stress views show no obvious signs of instability [13-15]. Each method of surgery treatment requires a level of experience. If the result of injury is partial tear of the SLIL, it will represent occult or predynamic instability [18,19]. For these injuries, most specialists recommend starting the treatment with splinting and/or casting [19,20]. Arthroscopic debridement with or without pinning can be an option for patients for whom the initial conservative treatment failed to succeed. With complete SLIL tears, immobilization does not reduce or prevent scapholunate dissociation [19]. Significant force occurs at the scapholunate interval on wrist loading. Options for acute management of these tears include direct repair with or without dorsal capsulodesis or arthroscopic debridement, reduction, and pinning. For chronic SL instabilities variable tendon reconstruction techniques and tenodesis have been described and tested with variable success, but when Brunelli and Brunelli [21] presented their method in 1995 it was a breakthrough in treatment of scapholunate instability, not because of its effectiveness in treatment of the scapholunate dissociation, but because it restricted and prevented the occurrence of rotatory subluxation of the scaphoid. It limited the flexion movements of the wrist, though, therefore three years later the procedure was modified by Abbeele [22] and became the best applicable technique in cases of dynamic scapholunate instability with rotatory subluxation of the scaphoid. Garcia-Elias described his own modification [15] of this procedure in 2006 and called it the three-ligament tenodesis for treatment of scapholunate dissociation. since 1995 only one case of avascular necrosis of the scaphoid [23] has been mentioned in available literature When logically reasoning about anatomical relationship between the scaphoid and the lunate, the new method presented here will restore better functioning of scapholunate ligament complex. Maintaining the distal part of the scaphoid in the axis of scapholunate complex by anchoring it, the free tendon graft will prevent rotatory subluxation of the scaphoid without the need of involvement of dorsal extrinsic ligaments as it is happening in modified Brunelli procedures. Since 1998, when Abbeele introduced his own modification to Brunelli procedure, we have found less than 400 cases treated with this technique [15,21-28]. The largest number of patients treated with this method were presented by Talwalker [25]. Most authors who revealed the results of implementing this technique unanimously agree that this is a method suitable for carefully selected cases; in addition better results were obtained in case of dynamic instabilities. Wrist range of motion got decreased in the range of 20% in dynamic instabilities to even 50% in static ones. The grip strength was also reduced even to 30% when compared to healthy wrists. Some publications informed of even better results than these after performing 4-corner fusion procedure [28].

Although this is not a clinical study, it shows that the mechanism of the new technique can restore the correct anatomical topography of scapholunate ligament complex with no deterioration of wrist movements. We also believe that in case of scapholunate instability with DISI deformity, the proposed modification of Brunelli technique could improve long-term results of surgical treatment.

## Conclusions

The proposed new technique was found to recreate anatomical forces and properties of scapholunate ligament and to prevent the occurrence of rotatory subluxation of the scaphoid. Modified Brunelli procedure proved to work very well in reducing the scapholunate gap and rotatory subluxation of the scaphoid, however it may limit the wrist movements because of involvement of dorsal extrinsic ligaments. Therefore the proposed new modification of Brunelli procedure could result in a more mobile wrist, with no evidence of scapholunate dissociation or scaphoid subluxation. In conclusion, we recommend the new technique and the new modified Brunelli procedure in treatment of scapholunate dissociation in both dynamic and static instabilities.

## Competing interests

We wish to confirm that there are no known competing interest associated with this publication and there has been no significant financial support for this work that could have influenced its outcome.

## Authors’ contributions

AE came up with the concepts of the new techniques, participated in cadaver dissections and technical measurements, performed statistical analysis and wrote the manuscript. JJ participated in the design of the study. BG was involved in technical measurements and participated in the study’s coordination. TJ and AD were involved in technical support and assistance. AD participated in cadaver dissections. All authors read and approved of the final manuscript.

## Pre-publication history

The pre-publication history for this paper can be accessed here:

http://www.biomedcentral.com/1471-2474/15/172/prepub
